# Effects of Ketamine and Tramadol As Adjuvants to Bupivacaine in Spinal Anesthesia for Unilateral Open Ovarian Cystectomy: A Randomized Controlled Trial

**DOI:** 10.7759/cureus.54776

**Published:** 2024-02-23

**Authors:** Ammar H Mahdi, Mohamed Kahloul, Myasar J Mohammed, Abbas K Mohammed

**Affiliations:** 1 Department of Anesthesia and Intensive Care, Faculty of Medicine of Sousse, University of Sousse, Sousse, TUN; 2 Department of Anesthesia and Intensive Care, Bilad Alrafidain University College, Baqubah, IRQ; 3 Department of Anesthesia and Intensive Care, Sahloul Teaching Hospital, Faculty of Medicine of Sousse, University of Sousse, Sousse, TUN; 4 Department of Anesthesia and Intensive Care, Balad Ruz General Hospital, Diyala Health Directorate, Iraqi Ministry of Health, Balad Ruz, IRQ

**Keywords:** duration of spinal analgesia, spinal adjuvants, ovarian cyst, tramadol, ketamine, spinal additives

## Abstract

Background: Spinal anesthesia offers numerous advantages and desirable features. However, it is associated with various side effects related to local anesthetic agents used. Reducing the dose of local anesthetic in spinal anesthesia can help minimize side effects but may lead to a diminished analgesic effect or failure of anesthesia. Therefore, adding an adjuvant may enhance the benefits while mitigating side effects.

Objective: This study aimed to evaluate the effects of ketamine and tramadol as adjuvants to bupivacaine on the duration of spinal analgesia. The objectives were to compare the three groups and prove their analgesic effects, safety, and superiority. The primary outcomes were the duration of spinal analgesia, as well as the onset and duration of both sensory and motor blocks. Secondary outcomes included the heart rate, mean arterial pressure, and the incidence of undesired effects such as nausea, vomiting, sedation, shivering, and postoperative headache.

Methods: In this double-blind randomized controlled trial, 120 female patients undergoing elective open unilateral ovarian cystectomy under spinal anesthesia were studied. The inclusion criteria included patients aged 16-45 years with a physical status classified as American Society of Anesthesiologists (ASA) class I and II. Patients were randomly allocated into three groups: group B (n=40) received only bupivacaine, group BK (n=40) received bupivacaine mixed with preservative-free ketamine, and group BT (n=40) received bupivacaine mixed with preservative-free tramadol.

Results: The mean duration of spinal analgesia, measured in minutes, showed significant differences (P < 0.001) between group BK (165 ± 4) and group B (170 ± 5). There was also a significant difference between group BT (313 ± 8) and group B (170 ± 5) (P < 0.001). Additionally, significant differences were observed between group BK (165 ± 4) and group BT (313 ± 8) (P < 0.001).

Conclusion: The administration of 25 mg of ketamine and 25 mg of tramadol as adjuvants to bupivacaine in spinal anesthesia significantly affected the postoperative duration of analgesia. Tramadol prolonged the duration of spinal anesthesia, while ketamine shortened it. The use of both adjuvants did not result in undesired effects.

## Introduction

Enhanced recovery after surgery (ERAS) protocols, which include locoregional anesthesia techniques, have significantly improved perioperative patient outcomes [1]. Among locoregional anesthesia techniques, spinal anesthesia is the most frequently utilized due to its well-established benefits. It offers superior control of postoperative pain, lowers the incidence of respiratory complications, particularly in cases involving difficult airway management or chronic respiratory conditions, and reduces the risk of postoperative cognitive dysfunction [2].

Despite its advantages, spinal anesthesia can lead to various side effects, such as hypotension, nausea, vomiting, itching, urine retention, post-dural puncture headache, total spinal anesthesia, neurological injury, spinal hematoma, and meningitis [3]. Hypotension induced by spinal anesthesia is a common complication that can have serious consequences, especially in patients with chronic cardiovascular diseases. The risk of hypotension is primarily dependent on the dosage of the local anesthetic used, highlighting the importance of reducing dosages without compromising the success of the anesthesia. In this context, the addition of adjuvants to local anesthetics may improve the efficacy of spinal anesthesia, decrease the required dosage of local anesthetics, and minimize side effects [4].

Opioids are the most commonly used adjuvants, enhancing neuroaxial anesthesia, reducing postoperative pain, and moderately prolonging the sensory block. Alpha2-adrenergic agonists, such as clonidine and dexmedetomidine, may expedite the onset and extend the duration of spinal anesthesia. However, the effectiveness of various adjuvants and the ideal adjuvant or dosage remains a subject of debate [5].

Tramadol, a weak opioid agonist, possesses sodium and potassium channel-blocking actions and ancillary actions, including the inhibition of norepinephrine and serotonin uptake [6]. It can block motor and nociceptive signals similarly to local anesthetics [7]. While the analgesic effects of tramadol as a central or peripheral analgesic are not fully understood, it is a selective agonist of μ-receptors. Tramadol also inhibits noradrenaline reuptake and enhances the release of both serotonin and noradrenaline [8]. Its monoaminergic activity augments the inhibitory function of descending pain pathways, leading to reduced nociceptive transmission at the spinal level [9].

Ketamine, known for its local anesthetic properties, is a noncompetitive antagonist of the N-methyl-D-aspartate (NMDA) receptor. It acts on multiple sites, including opioid receptors, monoaminergic receptors, voltage-sensitive calcium channels, and muscarinic receptors, in addition to its sodium channel-blocking local anesthetic actions. Systemic ketamine induces central summation in second-order pain neurons, thereby diminishing pain severity [4,10].

The primary outcomes were the onset and duration of both sensory and motor blocks, as well as the duration of spinal analgesia. Secondary outcomes included heart rate (HR), mean arterial pressure (MAP), nausea, vomiting, sedation, shivering, and postoperative headache.

This study aimed to evaluate the effects of ketamine and tramadol as adjuvants to hyperbaric 0.5% bupivacaine on the duration of spinal analgesia.

The study's hypothesis suggests that the utilization of ketamine and tramadol as adjuvant agents to hyperbaric 0.5% bupivacaine in spinal anesthesia results in a prolonged duration of spinal analgesia.

## Materials and methods

The study received approval from the Ethics Committee of the Diyala Health Directorate, Iraqi Ministry of Health (record number 75; April 2022). This prospective randomized, double-blind, controlled clinical trial was conducted at Balad-Ruz General Hospital in the Diyala Governorate, Republic of Iraq, from May 2022 to April 2023. A total of 120 female patients, aged 16-45 years and classified as American Society of Anesthesiologists (ASA) class I and II, scheduled for unilateral open ovarian cystectomy were included. Exclusion criteria included spinal deformity, bleeding tendency, people with mental health disorders, neurological diseases, history of scorpion bites, ASA classification of III or higher, and procedures exceeding 90 minutes.

The sample size was calculated using G*Power, version 3.1.9.4 (Heinrich Heine University Düsseldorf, Düsseldorf, Germany). The calculation was power-based, using the following values: α = 0.05, power (1-β) = 0.95, and effect size d = 0.82. These values were derived from data on the duration of spinal analgesia from a similar previous study [11], including median, standard deviation, and intended clinical significance to measure the effect size. (The effect size is a value that is calculated by the G-Power application and this is based on the result data of the mentioned similar previous study, assuming that the previous study fell into type 2 error.) The calculated sample size was 40 patients per study group; however, the study included 40 patients per group to account for potential dropouts.

**Figure 1:**
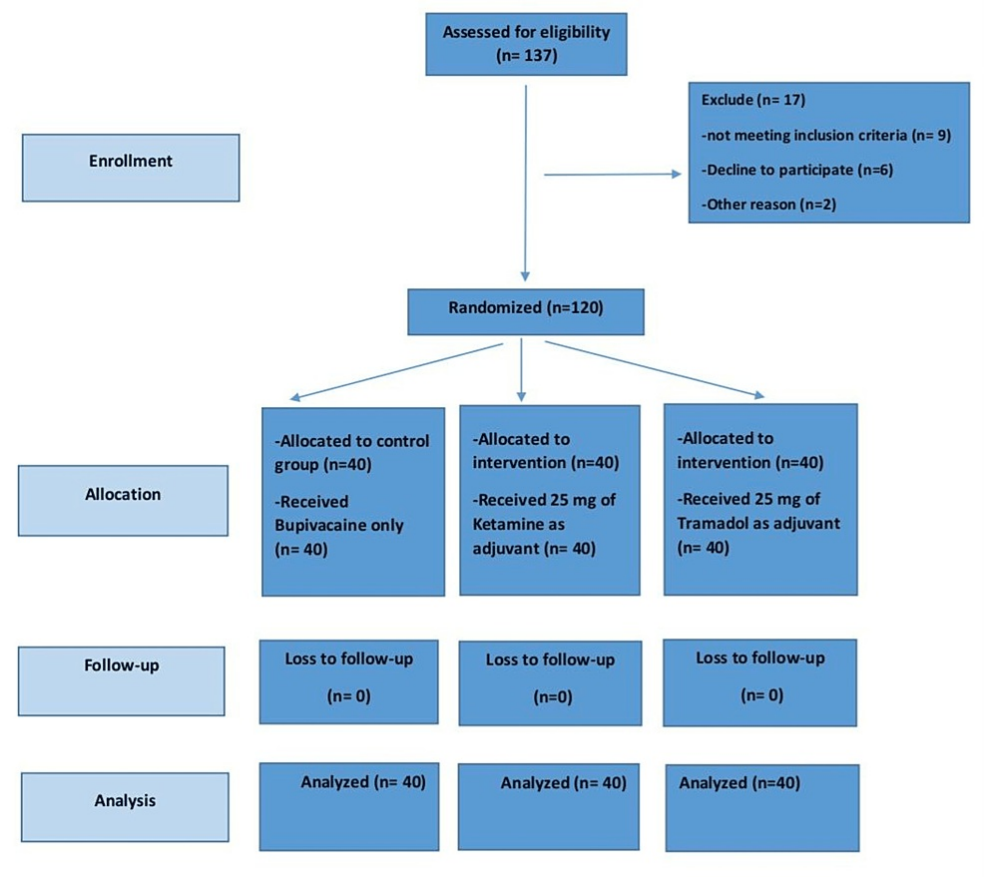
CONSORT flow diagram CONSORT: Consolidated Standards of Reporting Trials

Randomization was performed by an epidemiologist using Excel, version Professional Plus 2016 (Microsoft Corp., Redmond, WA, USA), employing a simple, parallel, double-blind randomization at a 1:1:1 ratio (Figure 1).

Informed consent was obtained from the patients or their relatives. Patients were randomly allocated into three groups: group B (n=40) received 2 mL of hyperbaric bupivacaine 0.5% mixed with 1 mL of normal saline. Group BK (n=40) received 2 mL of hyperbaric bupivacaine 0.5% mixed with 0.5 mL of preservative-free ketamine (25 mg), and group BT (n=40) received 2 mL of hyperbaric bupivacaine 0.5% mixed with 0.5 mL (25 mg) of preservative-free tramadol [11,12]. The volumes for groups BK and BT were completed with 0.5 mL of normal saline 0.9% to achieve a total volume of 3 mL.

Following an 8-hour fasting period, patients received a preload of normal saline. No premedication was administered. In the sitting position, spinal anesthesia was performed using a midline approach in an aseptic environment at the L3/L4 level, employing a G25 Quincke spinal needle. The study solution was injected at a relatively similar speed rate for all patients, who were then positioned supine. Vital signs, including HR, blood pressure, and oxygen saturation (SpO2), were monitored throughout the perioperative period, with a goal to maintain SpO2 above 95%. Oxygen was supplied at 3 L/min via nasal cannula. Blood pressure was maintained within 20% of baseline using crystalloids or ephedrine if systolic pressure fell below 90 mmHg.

Sensory block was assessed using a pinprick test, while motor block was evaluated using the modified Bromage scale (0: no loss, 1: unable to flex the hip, 2: unable to flex the knee, 3: unable to flex the ankle). Sedation was assessed every 10 minutes using a 4-point scale (1: awake, 2: drowsy but responsive to verbal commands, 3: responsive to physical stimulation, 4: unresponsive to verbal commands and physical stimulation). A score of 2 or higher was recorded as sedation.

The onset of sensory block was noted when the patient lost pinprick sensation, and the onset of motor block was recorded when a Bromage score of 3 was achieved. Both sensory and motor blocks were assessed at 30-second intervals to ensure precise monitoring of anesthesia progression. Monitoring for the duration of sensory block, motor block, and spinal analgesia was conducted at 5-minute intervals. The cessation of sensory block was indicated by the return of bilateral pinprick sensation at the S2 dermatome. Motor recovery was assumed when a Bromage score of 0 was noted. Patients were discharged pain-free after complete resolution of the motor block, stabilization of vital signs, and absence of nausea and vomiting.

During the preoperative evaluation, patients were instructed on assessing perioperative pain using a 10-point visual analog scale, with 0 representing no pain and 10 representing the worst pain imaginable. The duration of spinal analgesia was assessed starting from the administration of the medications until the visual analog scale score reached 1, 2, or 3, signifying the presence of mild pain. Data were entered into Microsoft Excel spreadsheets for analysis.

Statistical analysis

The collected data were revised, coded, and tabulated using the SPSS Statistics for Windows, version 25.0 (IBM Corp., Armonk, NY, USA). The data were presented, and appropriate analyses were conducted based on the type of data obtained for each parameter.

Descriptive statistics: Continuous data were summarized using mean values ± standard deviations to describe the central tendency and variability. Categorical data were evaluated as frequencies and percentages to detail the distribution of responses.

Analytical statistics: One-way ANOVA (analysis of variance) was used to determine the statistical significance of differences between the means of more than two study groups. ANOVA with repeated measures test was employed to evaluate the statistical significance of differences between parametric variables across more than two time periods. The chi-square test was applied to investigate the relationship between two qualitative variables. The Monte Carlo test was used to examine the relationship between two qualitative variables when the expected count was less than 5 in over 20% of cells.

## Results

Table 1 indicates that there were no significant differences among the groups concerning age, height, weight, and ASA classification.

**Table 1:** Comparison of personal data across all groups ASA, American Society of Anesthesiologists; SD, standard deviation; Min, minimum; Max, maximum; F, one-way ANOVA (analysis of variance) test statistic; χ², chi-square statistic; P-value, comparison between the three studied groups

	Group B bupivacaine n = 40		Group BK bupivacaine + ketamine n = 40		Group BT bupivacaine + tramadol n = 40		Test	p
Age (years)								
Mean ± SD	28± 6		28± 6		28± 6		F= 0.041	0.960
Min–Max	19–41		18–43		19–41			
Weight (kg)								
Mean ± SD	68± 8		68± 8		68± 8		F= 0.005	0.995
Min–Max	53–88		54–90		53–88			
Height (cm)								
Mean ± SD	165± 5		165± 4		166 ± 5		F= 0.124	0.884
Min–Max	157–179		157–178		157–179			
ASA	No.	%	No.	%	No.	%		
Class 1	33	82	32	80	30	75	ꭓ^2^= 0.707	0.702
Class 2	7	17	8	20	10	25		

Table 2 shows a significant difference in the onset time of sensory block among the three treatment groups (B, BK, and BT). Pairwise comparisons indicated that group B had a statistically significantly longer onset time compared to both groups BK and BT. However, no significant difference was found between groups BK and BT. Additionally, the mean duration of the sensory block differed significantly among the groups. Pairwise comparisons demonstrated that group BT (171 ± 8) had a significantly longer duration compared to group B (112 ± 7) and group BK (108 ± 12), while no significant difference was observed between groups B and BK.

**Table 2:** Comparison of sensory blockade, motor blockade, and duration of spinal analgesia between the three studied groups SD, standard deviation; Min, minimum; Max, maximum; F, one-way ANOVA (analysis of variance) test statistic; P1, comparison between the three studied groups; P2, comparison between bupivacaine and ketamine; P3, comparison between bupivacaine and tramadol; P4, comparison between ketamine and tramadol; *, significant

	Group B	Group BK	Group BT	F	P1	P2	P3	P4
	bupivacaine n = 40	bupivacaine + ketamine n = 40	bupivacaine + tramadol n = 40					
Sensory block								
Onset (min)								
Mean ± SD	4	3	3	75٫376	<0.001*	<0.001*	<0.001*	0٫203
Min–Max	3–6	2–4	2–4					
Duration (min)								
Mean ± SD	112 ± 7	108 ± 12	171± 8	524٫3	<0.001*	0٫158	<0.001*	<0.001*
Min–Max	100–130	90–140	155–185					
Motor block								
Onset (min)								
Mean ± SD	4	2	4	236٫5	<0.001*	<0.001*	<0.001*	<0.001*
Min–Max	4–5	2–3	3–5					
Duration (min)								
Mean ± SD	130 ± 11	120 ± 5	176 ± 11	368٫7	<0.001*	<0.001*	<0.001*	<0.001*
Min – Max	110 – 150	110 – 130	160 – 200					
Duration of spinal analgesia (min)								
Mean ± SD	170 ± 5	165 ± 4	313 ± 8	6879٫4	<0.001*	<0.001*	<0.001*	<0.001*
Min–Max	160–185	155–175	300–330					

In terms of motor block onset, significant differences were noted among the treatment groups. Group B had the longest mean onset time (4), followed by group BT (4), and group BK (2). The results also indicated significant differences in the duration of the motor block among the groups. Pairwise comparisons revealed significant differences between all group pairs, with group BT having the longest duration (176 ± 11), followed by group B (130 ± 11), and group BK having the shortest duration (120 ± 5).

Regarding the duration of spinal analgesia, significant differences were observed among the treatment groups. Pairwise comparisons showed significant differences between all group pairs. Group BT had the longest mean duration of spinal analgesia (313 ± 8), followed by Group B (170 ± 5), and Group BK had the shortest duration (165 ± 4).

Table 3 indicates that there were no statistically significant differences between the three groups in terms of undesired effects such as nausea, vomiting, headache, pruritus, and post-operative backache lasting three days. None of the participants in any group experienced dissociative effects. However, the incidence of shivering differed significantly among the groups. Pairwise comparisons revealed that group BT had a significantly lower incidence of shivering compared to group B (p = 0.026) and group BK (p = 0.026), while no significant difference was observed between groups B and BK (p = 1.000). Additionally, sedation was not reported in groups B and BT, but group BK had a statistically significantly higher incidence of sedation (p < 0.001).

**Table 3:** Comparison of undesired effects between the three studied groups χ², chi-square statistic; P1, comparison between the three studied groups; P2, comparison between bupivacaine and ketamine; P3, comparison between bupivacaine and tramadol; P4, comparison between ketamine and tramadol; *, significant

	Group B bupivacaine n = 40		Group BK bupivacaine + ketamine n = 40		Group BT bupivacaine + tramadol n = 40		Test	P1 B/K/T	P2 B/K	P3 B/T	P4 K/T
	No.	%	No.	%	No.	%					
Nausea											
No incidence	31	77	33	82	30	75	ꭓ^2^= 0.687	0.709	–	–	–
Incidence	9	22	7	17	10	25					
Vomiting											
No incidence	34	85	36	90	36	90	ꭓ^2^= 0.668	0.822	–	–	–
Incidence	6	15	4	10	4	10					
Headache											
No incidence	35	87	37	92	36	90	ꭓ^2^= 0.613	0.926	–	–	–
Incidence	5	12	3	7	4	10					
Pruritus											
No incidence	40	100	40	100	38	95	ꭓ^2^= 2.696	0.334	–	–	–
Incidence	0	0	0	0	2	5					
Shivering											
No incidence	34	85	34	85	40	100	ꭓ^2^= 8.007	0.023*	1.000	0.026*	0.026*
Incidence	6	15	6	15	0	0					
Sedation											
No incidence	40	100	24	60	40	100	ꭓ^2^= 48.0	<0.001*	<0.001*	–	<0.001*
Incidence	0	0	16	40	0	0					
Dissociative effects											
No incidence	40	100	40	100	40	100	–	–	–	–	–
Incidence	0	0	0	0	0	0					
Three days of postoperative backache											
No incidence	33	82	35	87	35	87	ꭓ^2^= 0.548	0.760	–	–	–
Incidence	7	17	5	12	5	12					

Table 4 demonstrates that at baseline, there was no significant difference (p ≥ 0.05) in the MAP among the three groups. However, at the 5-, 10-, 15-, 20-, 30-, 40-, 50-, 60-, 70-, 80-, and 90-minute time points, the p-values were all less than 0.05, indicating a significant difference in the mean MAP between the groups at these intervals. Specifically, the mean MAP in group B was statistically significantly lower than in groups BK and BT. Nonetheless, there was no statistically significant difference between groups BK and BT.

**Table 4:** Comparison of intraoperative MAP between the three studied groups MAP, mean arterial pressure, SD, standard deviation; Min, minimum; Max, maximum; F, one-way ANOVA test statistic; P1, comparison between the three studied groups; P2, comparison between bupivacaine and ketamine; P3, comparison between bupivacaine and tramadol; P4, comparison between ketamine and tramadol; *, significant

MAP (mmHg)	Bupivacaine n = 40	Bupivacaine + ketamine n = 40	Bupivacaine + tramadol n = 40	F	P1	P2	P3	P4
Baseline								
Mean ± SD	88± 6	89± 6	89 ± 7	0.124	0.884	–	–	–
Min–Max	73–103	73–103	73–103					
5 min								
Mean ± SD	79 ± 5	83± 6	83 ± 6	4.840	0.010*	0.022*	0.021*	1.000
Min–Max	69–90	70–95	69–95					
10 min								
Mean ± SD	79 ± 5	85 ± 5	85 ± 6	17.564	<0.001*	<0.001*	<0.001*	0.976
Min–Max	70–89	75–96	70–96					
15 min								
Mean ± SD	80 ± 3	86 ± 5	86 ± 6	16.130	<0.001*	<0.001*	<0.001*	0.994
Min–Max	73–88	75–96	75–96					
20 min								
Mean ± SD	81 ± 4	86± 7	86 ± 6	8.072	0.001*	0.002*	0.002*	0.999
Min–Max	73–89	72–98	72–98					
30 min								
Mean ± SD	82 ± 4	86 ± 7	86 ± 7	6.048	0.003*	0.013*	0.006*	0.964
Min–Max	70–89	73–98	70–98					
40 min								
Mean ± SD	82 ± 5	87± 7	87 ± 6	8.443	<0.001*	0.003*	0.001*	0.905
Min–Max	70–95	73–98	73–103					
50 min								
Mean ± SD	84 ± 6	89 ± 6	89± 6	7.840	0.001*	0.003*	0.002*	0.997
Min–Max	75–97	74–100	78–100					
60 min								
Mean ± SD	83 ± 4	89 ± 5	88 ± 5	18.600	<0.001*	<0.001*	<0.001*	0.946
Min–Max	74–93	78–98	78–98					
70 min								
Mean ± SD	83± 4	88 ± 6	88 ± 5	8.345	<0.001*	0.002*	0.002*	0.999
Min–Max	74–93	73–98	76–98					
80 min								
Mean ± SD	82± 4	87± 5	87 ± 5	10.169	<0.001*	0.001*	<0.001*	0.864
Min–Max	74–95	73–95	76–96					
90 min								
Mean ± SD	82± 5	87 ± 6	88 ± 5	10.441	<0.001*	0.001*	<0.001*	0.808
Min–Max	70 – 93	73 – 98	74– 95					

**Figure 2:**
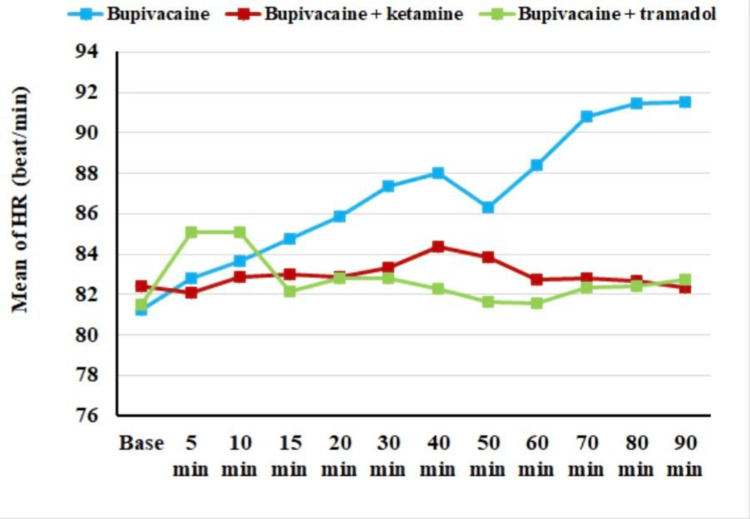
Comparison of HR among the three studied groups HR: heart rate

Figure 2 demonstrates that there was no significant difference (p ≥ 0.05) in the mean HR between the three groups at baseline, 5, 10, 15, and 20 min. However, the p-values for comparing the three groups at the 30-, 40-, 50-, 60-, 70-, 80-, and 90-minute time points were all less than 0.05, which means that there was a significant difference in the mean HR between the three groups at these time points. Among 40 and 50 minutes, the mean HR was statistically significantly higher in the bupivacaine group than in the bupivacaine + tramadol group. However, there was no statistically significant difference between the other two groups. Regarding HR at 30, 60, 70, 80, and 90 minutes, the results showed that the bupivacaine group was statistically significantly higher than the ketamine and tramadol groups. However, there was no statistically significant difference between the ketamine and the tramadol groups.

## Discussion

Ketamine is known to produce analgesic effects following epidural, caudal, or intrathecal administration [13]. These effects are mediated through various mechanisms: ketamine binds to opiate receptors and interacts with cholinergic, adrenergic, and 5-hydroxytryptamine systems [14]. It inhibits the excitation of central neurons by NMDA [15] and prevents action potential conduction by affecting sodium and potassium channels in nerve membranes, thus exhibiting local anesthetic properties [16].

In this study, the addition of ketamine to bupivacaine for spinal anesthesia resulted in a shortened duration of spinal analgesia, as well as reduced onset and duration of sensory and motor blocks. These findings align with those of El-Fawy and Kamal [11], who observed that ketamine reduced the onset time and duration of sensory and motor blocks, as well as the overall duration of analgesia. Similarly, Kathirvel et al. found that ketamine shortened the duration of motor blockade when added to intrathecal bupivacaine, compared to bupivacaine alone [17]. Yanli and Eren found that extradural ketamine decreased the onset time of motor blockade, and Gantenbein et al. noted that ketamine significantly enhanced the local anesthetic activity of bupivacaine, possibly due to ketamine’s inhibitory effect on bupivacaine metabolism [18,19]. Consequently, the combination of ketamine with bupivacaine may be advantageous for day-case procedures, as it aligns with the objectives of such procedures. Contrasting these results, Khezri et al. reported that intrathecal ketamine with bupivacaine extended intraoperative anesthesia, though this discrepancy may be attributed to their study’s focus on pregnant women [20]. Weir and Fee observed behavioral changes associated with high doses of ketamine, whereas this study investigated the effects of smaller doses of additives [21].

Tramadol, a centrally-acting analgesic agent with a terminal elimination half-life of 5.5 hours, provides clinical analgesia for up to 10 hours following epidural administration [22]. It primarily stimulates the µ-opioid receptors and, to a lesser extent, the δ and κ receptors. Additionally, tramadol facilitates spinal inhibition of pain by reducing the reuptake of norepinephrine and serotonin. Although tramadol’s analgesic potency is one-fifth that of morphine, it is associated with less respiratory depression and pruritus. Other studies have suggested that tramadol may exert local anesthetic effects on peripheral nerves [23]. The addition of tramadol significantly prolonged the duration of spinal analgesia beyond that achieved with bupivacaine alone or with bupivacaine plus ketamine. These results are consistent with those reported by Chakraborty et al. and Kaabachi et al. [22,24]. In the BK group, 16 out of 40 patients reached a sedation score of 2 on the four-point scale, whereas no sedation was observed in the B and BT groups. This effect is attributed to ketamine’s solubility properties, which facilitate its intravascular absorption. Vincenzi et al. used 20 mg of ketamine as an intrathecal adjuvant for sedation [25]. Pruritus was noted in two patients in the BT group (n=40), likely related to the activation of opioid µ receptors [26].

Spinal anesthesia can lead to hypotension as a physiological consequence of sympathetic blockade [27]. The MAP decreased in the BK and BT groups, albeit to a lesser extent than in the B group. The findings of Kathirvel et al. [17] align with this study, showing that patients receiving bupivacaine alone had significantly lower mean systolic and diastolic blood pressures compared to those receiving adjuvant ketamine. Similarly, Prasad et al. demonstrated a reduced incidence of hypotension in patients who received adjuvant tramadol compared to those given bupivacaine alone [28]. In contrast, El-Fawy and Kamal, and Togal et al. found no significant difference between using bupivacaine alone and using ketamine as an adjuvant [11,29]. Alhashemi and Kaki also found no differences in systolic and diastolic pressures when using bupivacaine alone or with tramadol as an adjuvant [30].

The initial HR baseline tends to be consistent across all patients, indicating the anesthetists' efforts to maintain HR within the normal range for administering spinal anesthesia. In the first 10 minutes, HR increased in all three groups, but from 10 to 90 minutes, the HR in both adjuvant groups notably decreased compared to the bupivacaine group, remaining within or close to the normal range. Conversely, the bupivacaine group exhibited higher HR values. These variations in HR are justifiable, representing compensatory responses reflecting the immediate decrease in MAP following anesthesia induction due to sympathetic blockade, with this effect gradually diminishing. Except for the bupivacaine group, MAP remained lower than in the other groups throughout the observation period. This result is not compatible with Kamal and El-Fawy, and Togal et al. [11,29]. No research aligns with our study in terms of HR findings.

Neuraxial anesthesia leads to significant vasodilation, resulting in heat loss and a subsequent reduction in body core temperature. Intraoperative shivering during neuraxial anesthesia, even at a constant ambient temperature, can be triggered by various mechanisms. These include body core hypothermia due to heat loss and redistribution, heat loss exceeding metabolic heat production, and the anesthetic-induced inhibition of thermoregulatory control, both centrally and peripherally. This study observed that shivering occurred in six patients in each of the B and BK groups, while no patients in the BT group experienced shivering. The anti-shivering effect of tramadol is likely mediated through its opioid activity, as well as its serotonergic and noradrenergic actions.

Across all three groups, there were no statistically significant differences in the occurrence of nausea, vomiting, and headache during the three postoperative days. Notably, no cases of anesthesia failure were recorded.

## Conclusions

The pain-free period ranked from longest to shortest as follows: BT (bupivacaine + tramadol) Group (310 minutes) > B (bupivacaine) Group (170 minutes) > BK (bupivacaine + ketamine) Group (165 minutes). Adding ketamine and tramadol to bupivacaine is safe, provides better hemodynamic stability than using bupivacaine alone, and with no observed adverse effects. Further investigation is warranted to ascertain optimal dosage, administration method, and cost implications.
